# The first “London Declaration”: The Commonwealth and its neglected tropical diseases

**DOI:** 10.1371/journal.pntd.0005321

**Published:** 2017-04-27

**Authors:** Peter J. Hotez, Ashish Damania, Aparna Barua, Jeffrey Stanaway

**Affiliations:** 1 Texas Children’s Hospital Center for Vaccine Development, National School of Tropical Medicine at Baylor College of Medicine, Houston, Texas, United States of America; 2 Department of Biology, Baylor University, Waco, Texas, United States of America; 3 Center for Health and Biosciences, James A Baker III Institute for Public Policy, Rice University, Houston, Texas, United States of America; 4 Scowcroft Institute of International Affairs, Bush School of Government and Public Service, College Station, Texas, United States of America; 5 Sabin Foundation Europe, London, United Kingdom; 6 Institute for Health Metrics and Evaluation, University of Washington, Seattle, Washington, United States of America; University of Washington, UNITED STATES

**Fig 1:**
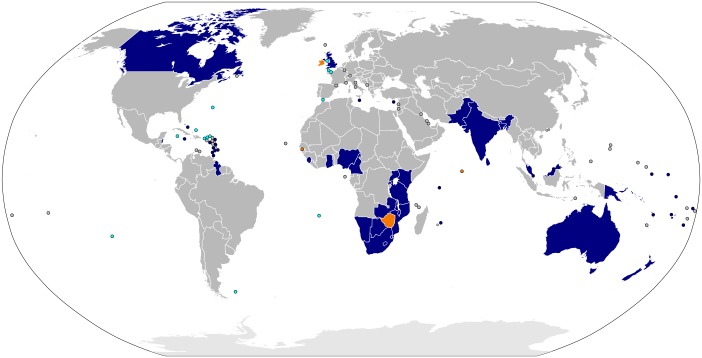
Map of the Commonwealth of Nations. By Wikimedia Commons user Rob984. Available here: https://en.wikipedia.org/wiki/Commonwealth_of_Nations#/media/File:Member_states_of_the_Commonwealth_of_Nations.svg.

The Commonwealth, also known as the Commonwealth of Nations, is a voluntary union of independent sovereign states with historic ties to the British Empire and was formerly known as the British Commonwealth [1]. Today, the Commonwealth comprises 52 nations, the most populous being Bangladesh, India, and Pakistan in South Asia, and Nigeria, South Africa, Tanzania, and Uganda in sub-Saharan Africa (Fig 1). More than 2 billion people representing almost one third of the world’s population live in Commonwealth nations [1].

Some ascribe the origins of the modern Commonwealth to a 1949 London Declaration issued by the fourth Commonwealth Prime Ministers’ Conference. The declaration, represented by the United Kingdom, Canada, Australia, New Zealand, South Africa, India, Pakistan, and Ceylon (Sri Lanka), affirmed the Commonwealth's constituent members’ independence from the UK. This was a marked change and resulted from an agreement to continue India’s membership post-independence in 1947 [2]. It also highlighted the renewed member states’ commitment of “freely cooperating in the pursuit of peace, liberty, and progress” [2]. Today, Queen Elizabeth II serves as the Head of the Commonwealth, with a Commonwealth Secretariat based in London. The Secretariat is overseen by a Secretary General and is divided into three divisions: political, social development, and corporate and economic [1]. The current and sixth Commonwealth Secretary General is the Rt Hon Patricia Scotland QC, a former British parliamentarian who held the positions of Minister of State for the Home Office and Attorney General (2007–2010) and served as the Chief Legal Advisor to the Crown and its government in England and Wales. Born in Dominica, she is the first woman to serve in this role [3].

The framework of cooperation of the Commonwealth of Nations provides a useful mechanism for global efforts to control or eliminate neglected tropical diseases (NTDs), including the 18 NTDs currently identified by the World Health Organization.

Overall, based on Global Burden of Disease (GBD) Study 2015 data [3], almost one half of the approximately 23 million global disability-adjusted life years (DALYs) from NTDs are found in the Commonwealth, but for some specific diseases such as leishmaniasis, trachoma, and lymphatic filariasis (LF) almost two thirds or more of the DALYs occur in the Commonwealth of Nations (Table 1). The only NTDs with a negligible burden in the Commonwealth are Chagas disease, which is mostly found in Latin America and the United States, and food-borne trematodiases, which are found predominantly in China and Southeast Asia.

**Table 1:** Neglected Tropical Disease (NTD) Disability-Adjusted Life Years (DALYs) globally and in Commonwealth Nations, 2015.

Disease	Global DALYs^a^	DALYs in Commonwealth of Nations^a^	Percentage of global DALYs occurring in Commonwealth of Nations
Leishmaniasis	1,418,933	1,041,548	73.4
Trachoma	279,236	178,275	63.8
Lymphatic filariasis	2,074,955	1,246,196	60.1
Rabies	931,556	538,783	57.8
Cysticercosis	303,632	153,876	50.7
Intestinal nematode infections	3,378,336	1,698,401	50.3
Schistosomiasis	2,613,310	1,244,634	47.6
Other NTDs	6,460,962	2,824,941	43.7
Yellow fever	329,755	115,795	35.1
Cystic echinococcosis	172,630	56,076	32.5
Onchocerciasis	1,135,703	360,870	31.8
Dengue	1,892,234	591,114	31.2
African trypanosomiasis	202,438	32,030	15.8
Chagas disease	236,137	109	<0.1
Food-borne trematodiases	1,686,463	0	0.0
Total	23,116,283	10,082,647	43.6

^a^Source of DALYs from the Global Burden of Disease (GBD) Study 2015 website and [3].

The finding of widespread NTDs, especially LF and trachoma, among the Commonwealth of Nations has important policy implications. First, the Rt Hon Priti Patel, the UK Secretary of State for International Development who was appointed in 2016, has voiced the importance of tying the UK government’s Department for International Development (DFID) overseas development assistance to cost-effective solutions and to those having an impact relevant to the citizens of the UK [4]. In 2016, she told *The Guardian* that her “approach will be built on some core conservative principles: That the way to end poverty is wealth creation” [4]. Indeed, NTDs are known to be stealth forces that trap people in poverty because of their long-term deleterious effects on worker productivity, child development, and women’s health [5], such that NTD control and elimination efforts represent highly effective antipoverty measures.

Yet another key reason is that LF and trachoma have the potential to be eliminated over the next decade by expanding access to low-cost or donated drugs. Indeed, this activity is central to a different London Declaration, the 2012 London Declaration on NTDs [6]. The finding that much of the world’s LF and trachoma cases are found in the Commonwealth of Nations provides an impetus to make NTD elimination an essential element to achieving its core mission: improving the well-being of all Commonwealth citizens and advancing their shared interests globally. In the case of leishmaniasis, the high disease burden found in Commonwealth nations reflects its high prevalence and incidence in South Asia and highlights the need for new tools—better drugs, better diagnostics, and vaccines—to achieve global elimination efforts.

Together with DFID, the Commonwealth Secretariat has a unique opportunity to elevate the profile of NTD elimination efforts by including NTDs on the agenda of the annual Commonwealth Health Ministers Meeting in Geneva and the biannual Commonwealth Heads of Government Meeting, to be hosted by the UK in 2018. As we mark the fifth anniversary of the London Declaration on NTDs this year, we increasingly recognize the urgent need for increased domestic country leadership across NTD programs and research investments. Without this political prioritization, human resources and finances at current levels will not be enough to meet the social and economic challenges these debilitating diseases present to the world’s poorest people. Many of the Commonwealth nations host enormous research and development capabilities that could produce new generation drugs, diagnostics, and vaccines (control tools) for NTDs. Ultimately, the Commonwealth of Nations and its Secretariat, DFID, and partner organizations could take on a powerful role in eliminating these ancient scourges of the world’s poor and perhaps the most common diseases of people living in poverty. In doing so, the Commonwealth and DFID could actually lift hundreds of millions of people out of poverty.
